# Antimicrobial resistance: progress and challenges in antibiotic discovery and anti‐infective therapy

**DOI:** 10.1111/1751-7915.13945

**Published:** 2021-10-05

**Authors:** Tino Krell, Miguel A. Matilla

**Affiliations:** ^1^ Department of Environmental Protection Estación Experimental del Zaidín Consejo Superior de Investigaciones Científicas Prof. Albareda 1 Granada 18008 Spain

## Abstract

The alarming rise in the emergence of antimicrobial resistance in human, animal and plant pathogens is challenging global health and food production. Traditional strategies used for antibiotic discovery persistently result in the re‐isolation of known compounds, calling for the need to develop more rational strategies to identify new antibiotics. Additionally, anti‐infective therapy approaches targeting bacterial signalling pathways related to virulence is emerging as an alternative to the use of antibiotics. In this perspective article, we critically analyse approaches aimed at revitalizing the identification of new antibiotics and to advance antivirulence therapies. The development of high‐throughput *in vivo*, *in vitro* and *in silico* platforms, together with the progress in chemical synthesis, analytical chemistry and structural biology, are reviving a research area that is of tremendous relevance for global health.

## Graphical abstract

The rise in the emergence of antimicrobial resistance in pathogenic bacteria urges the development of novel approaches to combat pathogenic bacteria. Here, we analyze major strategies aimed at the identification of novel natural product‐based antibiotics from microbial sources and to advance anti‐infective therapy strategies targeting signal transduction pathways.

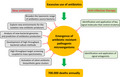

## Introduction

We are facing difficult times economically, ecologically and health‐wise. Fortunately, we live in a scientific age that has shown its potential with the development of several COVID‐19 vaccines in record time, with numerous additional vaccines in different stages of development or applying for a licensing (Brüssow, 2021), reflecting the capacity of our society to overcome a major health crisis.

One of the greatest challenges of our time is the increasing emergence antimicrobial resistance (AMR). AMR microbes are currently causing 2.8 million infections and more than 35 000 deaths per year only in the United States (Centers for Disease Control and Prevention, 2019), as well as 700 000 annual deaths worldwide (Miethke *et al*., 2021). This global health problem does not discriminate between economic and social levels and affects low‐, middle‐ and high‐income countries (World Health Organization, 2015). Dramatically, if prompt actions are not taken, current estimates indicate that 10 million people will annually die in 2050 as a result of AMR infections (Dadgostar, 2019), surpassing cancer as the leading cause of mortality at a global scale (O’Neil, 2016). AMR will lead to longer hospital stays, increased second‐line treatments and treatment failure. It was estimated that treatment of AMR infections can incur a cost of up to USD 50 000 per patient and episode, and that USD 20 billion per year are necessary to palliate AMR in the United States (Naylor *et al*., 2018; Dadgostar, 2019). AMR not only impacts on animal and human health but also on agricultural production. Crop production must increase by more than 60% with respect to the current production to feed an expected population of 10 billion people in 2050 (Bhatta *et al*., 2021). Nevertheless, according to the Food and Agriculture Organization of the United Nations (FAO), plant pathogens are responsible for losses of up to 40% of the annual global crop production, corresponding to a value of USD 290 billion. Traditionally, fighting plant pathogens has been achieved by the use of chemical pesticides that have resulted in the emergence of resistances that in turn hamper the effective treatment of these phytopathogens (Sundin and Wang, 2018).

Thus, urgent actions need to be taken to effectively control human, animal and plant AMR pathogens. Scientifically, this aspect is reflected in the fact that (i) ~ 16% of academic projects are focused on drug discovery (Bem *et al*., 2015) and (ii) ~ 70% of the projects in the preclinical antibacterial pipeline are aimed at the identification of antibiotics that interact with novel targets (Theuretzbacher *et al*., 2020). Unfortunately, these research lines are primarily conducted by academia and are generally underfunded. Consequently, future strategies will require an increased cooperation between academic and industrial sectors in order to improve the success rates of novel antibiotic discovery (Miethke *et al*., 2021). This will require to (re‐) attract pharmaceutical companies to invest into antibiotic research; aspect that will involve governments and policymakers across the globe to quickly legislate means that lessens the enormous capital risks of antibiotic research and development funding.

Although there is a desperate need for the discovery and production of novel antibiotics, the concept of anti‐infective therapy is an attractive alternative to antibiotics (Cegelski *et al*., 2008; Bjarnsholt *et al*., 2013; Allen *et al*., 2014). Anti‐infective therapy is based on targeting molecular mechanisms that lead to disease but that do not interfere with bacterial growth. In other words, this strategy aims at disarming rather than killing bacteria. Bacteria contain a wide range of signal transduction systems such as transcriptional regulators, two‐component systems, chemosensory pathways, phosphoenolpyruvate:sugar phosphotransferase systems, adenylate cyclases and cAMP phosphodiesterases, diguanylate cyclases and c‐di‐GMP phosphodiesterases, extracytoplasmic function sigma factors and Ser/Thr/Tyr protein kinases and phosphatases (Galperin, 2018). These systems sense different types of signals and generate different responses such as transcriptional regulation, control of second messenger levels or chemotaxis. Through the concerted action of these systems, bacteria are able to optimally adapt to their present environment or move chemotactically towards more favourable niches. In the context of anti‐infective therapy, the interference with such signal transduction pathways has the promise to become an efficient strategy to fight pathogenic bacteria (Calvert *et al*., 2018; Miethke *et al*., 2021).

In this perspective article, we focus on current and future approaches aimed at identifying novel natural product‐based antibiotics from microbial sources as well as in the development of anti‐infective therapy strategies that target microbial signal transduction pathways relevant for virulence.

## The need to diversify microbial isolation sources to improve antibiotic discovery rates

Historically, Actinobacteria have been the main source of antimicrobial compounds and around two thirds of all clinically used antibiotics are produced by members of this phylum (van Bergeijk *et al*., 2020). Remarkably, the analysis of the new chemical entities identified over the last 40 years revealed that natural product‐based compounds continue to be the main source of antibacterial compounds (Newman and Cragg, 2020). However, the probability of identifying new antibiotics from Actinobacteria using classical methods (i.e. bioactivity‐guided isolation through the screening of environmental isolates) is very low and has been estimated at less than one in a million (van Bergeijk *et al*., 2020), which is urging the scientific community to advance new experimental and *in silico* alternatives to improve discovery rates. In this regard, there is much controversy as to whether efforts to identify new natural product‐derived antibiotics should continue to be focused on the screening of soil Actinobacteria or whether the phylogenetic range should be broadened and new or poorly characterized taxa included (Lewis, 2020; Gavriilidou *et al*. 2021). In this context, the advances in the development of genome mining methods for the prediction of natural products biosynthetic genes from genome sequence data are starting to provide some answers to these questions. Indeed, the success of improved genome mining approaches has increased the discovery rate of novel bioactive secondary molecules (Blin *et al*., 2021; Medema *et al*., 2021). The enormous potential of these *in silico* strategies is reflected in a recent survey of ~ 170 000 bacterial genomes and ~ 10 000 bacterial metagenome assembled genomes which has been deposited in BioRxiv (Gavriilidou *et al*. 2021). Major conclusions from this work are: (i) only 3% of the bacterial biosynthetic potential for natural product synthesis has been explored experimentally and (ii) although actinobacterial strains showed the highest natural product biosynthetic diversity, less studied taxa also exhibit strong potential for the synthesis of new antibiotics. Consequently, efforts should focus on exploring new environments and ecological niches as alternative isolation sources of new antibiotic producers such as the human microbiome (Donia *et al*., 2014), sponge microbiota (Wilson *et al*., 2014) or rhizospheric soils (Crits‐Christoph *et al*., 2018; Carrión *et al*., 2019), as well as on expanding the taxonomic range of potential antibiotics producing bacteria. For instance, the enormous potential of this experimental diversification has been exemplified by the reconstruction of bacterial genomes from grassland soil metagenomes (Crits‐Christoph *et al*., 2018). This seminal study allowed the identification of several under‐investigated bacterial phyla like Gemmatimonadetes, Verrucomicobia, Acidobacteria and Rokubacteria as prolific sources of secondary metabolites of the non‐ribosomal peptide, polyketide and terpene classes, among others. Outstandingly, most of the natural product biosynthetic gene clusters from these organisms were novel and some of the identified bacteria dedicated up to 14% of their genomes to the production of secondary metabolites (Crits‐Christoph *et al*., 2018), which highlights the relevance of taxa alternative to the Actinobacteria phylum as sources of novel antibiotics.

Whereas genome mining methods lay the groundwork for the identification of novel antibiotics, advanced analytical chemistry is required to confirm the identity of the desired molecule, an aspect that is hampered when the secondary metabolites are produced at low titres. To improve the efficiency of antibiotic discovery, great progress is being made in the development of algorithms that allow to establish associations between biosynthetic gene clusters and the structure of corresponding bioactive molecule, as well as to predict the function of the molecule of interest from available sequence information (Medema *et al*., 2021). The advance of these and additional technologies will help prioritize among candidate antibiotic biosynthetic clusters and to rapidly improve performance of antibiotic discovery in the short‐term future.

## Microfluidics approaches to enhance bacterial cultivability aimed at antibiotic discovery

Although genome mining approaches permit access to untapped secondary metabolites, one of the main constraints encountered in the field of antibiotic discovery at present is the fact that many potential source bacteria are recalcitrant to cultivation. Indeed, current data indicate that < 1% of environmental bacteria are cultivable under standard growth conditions (Lloyd *et al*., 2018), which hinders the identification of novel antibiotics. In order to improve bacterial cultivability, current strategies are mainly focused on developing high‐throughput culture methods (Lagier *et al*., 2018). In addition, alternative approaches permit bacterial growth in their natural environment using, for example, the isolation chip (iChip) technology, which is based on the use of semi‐permeable membranes that permit free diffusion of specific nutrients and growing factors from the environment into the bacterial culture (Lewis, 2020; Miethke *et al*., 2021). In the context of searching for specific culture conditions for the growth of uncultured antibiotic‐producing bacteria, extraordinary progress has been made with the development of high‐throughput microfluidics approaches (Hengoju *et al*., 2020; Matilla, 2021). Within this expanding research field, microfluidics permits partitioning complex bacterial communities into droplets containing a single cell. These droplets function as micro‐reactors where physiological and biochemical parameters can be monitored and optimized. Remarkably, such systems have successfully identified culture conditions for novel antibiotics producers (Hengoju *et al*., 2020; Matilla, 2021). For instance, droplet‐based systems mimicking environmental conditions *in situ* have enabled the cultivation of a higher microbial diversity as compared with conventional culture techniques, including prolific antibiotic producers (Mahler *et al*., 2021). Importantly, droplet microfluidics coupled to mass spectrometry has been effectively used for the detection and monitoring of secondary metabolites in bacteria (Hengoju *et al*., 2020) and the rapidly improving performance of analytical chemistry techniques is expected to provide a major boost to microfluidics‐based approaches for antibiotic discovery.

## Activation of silent antibiotic biosynthetic gene clusters

The genetic capacity for the synthesis of secondary metabolites or the possibility to culture the source microorganism outside its natural habitat does not generally guarantee the production of the desired metabolite. This lacking synthesis is primarily due to the intricate regulatory network that controls the expression of secondary metabolite biosynthetic gene clusters, which results in most of them being silent under standard culture conditions. Consequently, these cryptic secondary metabolites represent a hidden source of bioactive compounds with antibiotic properties.

**Fig. 1:**
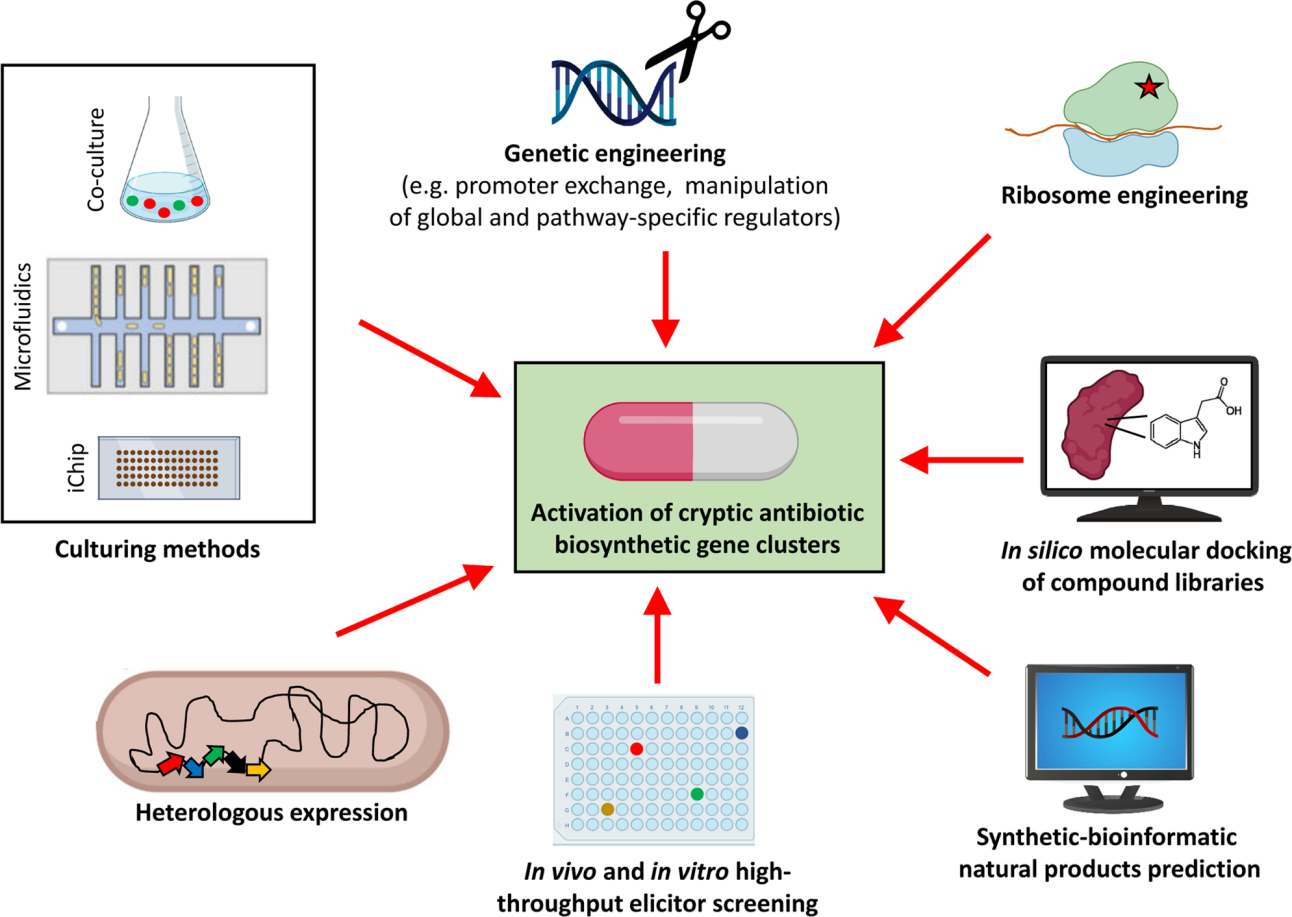
Major *in vivo*, *in vitro* and *in silico* approaches aimed at the activation of cryptic natural product‐based antibiotics.

A range of experimental approaches aimed at activating cryptic gene clusters, such as co‐culture methods, heterologous pathway expression, ribosome engineering, promoter exchange, mutation and overexpression of regulatory genes, have proven to be effective in the identification of cryptic antibiotics and are detailed in a number of comprehensive reviews (Rutledge and Challis, 2015; Okada and Seyedsayamdost, 2017; Mao *et al*., 2018; Kang and Kim, 2021; Scherlach and Hertweck, 2021) (Fig. 1). Additionally, culture‐independent strategies that rely on the chemical synthesis of target compounds based on bioinformatics predictions, named synthetic‐bioinformatic natural products, are also being developed to access silent secondary metabolites (Scherlach and Hertweck, 2021) (Fig. 1). However, we would like to pay here special attention to the intricate regulatory cascades that control secondary metabolites production and the tremendous possibilities derived from the characterization of these regulatory pathways for the activation of cryptic metabolites.

The biosynthesis of secondary metabolites is energetically costly and the expression of the corresponding biosynthetic gene clusters is tightly regulated by global and pathway‐specific regulators in response to external stimuli (Okada and Seyedsayamdost, 2017; van der Heul *et al*., 2018; van Bergeijk *et al*., 2020; Matilla *et al*., 2021). The identification of these regulators is key to understand the fine‐tuned expression of secondary metabolites, and high‐throughput functional genomics approaches are being developed to uncover complex bacterial regulatory networks (Smith *et al*., 2021). Many of the regulatory proteins that control antibiotic production sense different signal molecules (Matilla *et al*., 2021), and the identification of these signals is of major importance to activate cryptic biosynthetic cluster for antibiotic discovery. However, the nature of most of these chemical elicitors is unknown, and this aspect is a major bottleneck in the study of bacterial signal transduction that is of particular relevance in the antibiotic discovery field. To overcome this difficulty, different *in vivo* and *in vitro* high‐throughput screening approaches of extensive chemical libraries are being developed (Fig. 1); some of which were shown to be effective in the activation of cryptic antibiotics (Mao *et al*., 2018; Zhang and Seyedsayamdost, 2020; Scherlach and Hertweck, 2021). Importantly, these screenings can be coupled to mass spectrometry‐based metabolomics to facilitate natural products identification (Zhang and Seyedsayamdost, 2020). As a proof of concept, we have successfully screened *in vitro* a compound library of ~ 1700 small molecules to identify several phytohormones as ligands of an antibiotic‐specific transcriptional regulator in a plant‐associated bacterium that has resulted in the identification of indole‐3‐acetic acid as a modulator of the production of the antibiotic andrimid (Matilla *et al*., 2018). Additionally, advances in integrated computational approaches for antibiotic discovery such as *in silico* docking of virtual compound libraries and molecular dynamics simulations (Lans *et al*., 2020; Macalino *et al*., 2020) can be used to identify ligands of key transcriptional regulators and sensor kinases to improve the efficiency in the identification of chemical elicitors of cryptic antibiotics biosynthesis (Fig. 1). Notably, complementary to the *in vivo* and *in vitro* approaches described above aimed at activating cryptic biosynthetic clusters, additional high‐throughput proteomics‐based strategies are being implemented for *de novo* identification of antibiotic targets (Martin *et al*., 2020). Taken together, the combination of different *in vivo*, *in vitro* and *in silico* high‐throughput screenings will facilitate the discovery of new antibiotic compounds with novel mechanisms of action in the near future.

## Anti‐infective therapy as alternative for the treatment of bacterial infections

The large array of bacterial signal transduction systems illustrates well their importance for bacteria to adapt to their environment and to move to more favourable environmental niches. In the context of fighting pathogenic bacteria, there are a number of examples that illustrate that the interference with signalling processes can be achieved by (i) the application of a key signal molecule that regulates bacterial virulence, (ii) the use of signal antagonists that bind to sensor proteins but do not induce downstream signalling or (iii) the interference with the signalling cascade. An advantage of such strategies is that they typically do not kill bacteria or interfere with growth, preventing the selection of AMR mutants.

The first strategy is best illustrated by *Pseudomonas aeruginosa* – a human pathogen classified by the World Health Organization as a priority 1 (critical) pathogen for development of novel antibiotics (Tacconelli *et al*., 2018). It was shown that low concentrations of inorganic phosphate (Pi) induce a lethal phenotype during intestinal colonization (Zaborin *et al*., 2009). Pi is perceived by the PhoR/B two‐component system (Peng *et al*., 2017), and transcriptomics studies showed that a modest reduction in the Pi concentration, that is, 1 mM to 0.2 mM, caused increased transcript levels of many virulence genes (Bains *et al*., 2012). Via animal experimentation it was shown that Pi administration reduces *P. aeruginosa* infection (Long *et al*., 2008). In general, surgical interventions cause a significant decrease in the Pi concentration. A mouse model was established in which a surgical intervention was followed by a challenge with *P. aeruginosa* (Long *et al*., 2008). It was found that the challenge of the mouse control group resulted in an elevated lethality. However, when mice challenged with *P. aeruginosa* had previously received oral Pi, the survival rate was significantly higher. The potential of such approach to reduce bacterial virulence is furthermore illustrated by the facts that Pi is cheap and a natural compound that does not cause any undesired side‐effects.

Unfortunately, the knowledge on the identity of signal molecules that determine bacterial virulence is still rather scarce. For example, *P. aeruginosa* contains 64 sensor kinases of which the large majority were found to be involved in virulence (Francis *et al*., 2017). However, the signals that control kinase activity have only been determined for a handful of proteins (Francis *et al*., 2017). Therefore, the precise knowledge of virulence‐related signal molecules will offer elegant strategies to interfere with infectious processes.

## Antagonists targeting signal transduction systems in anti‐infective therapy

**Fig. 2:**
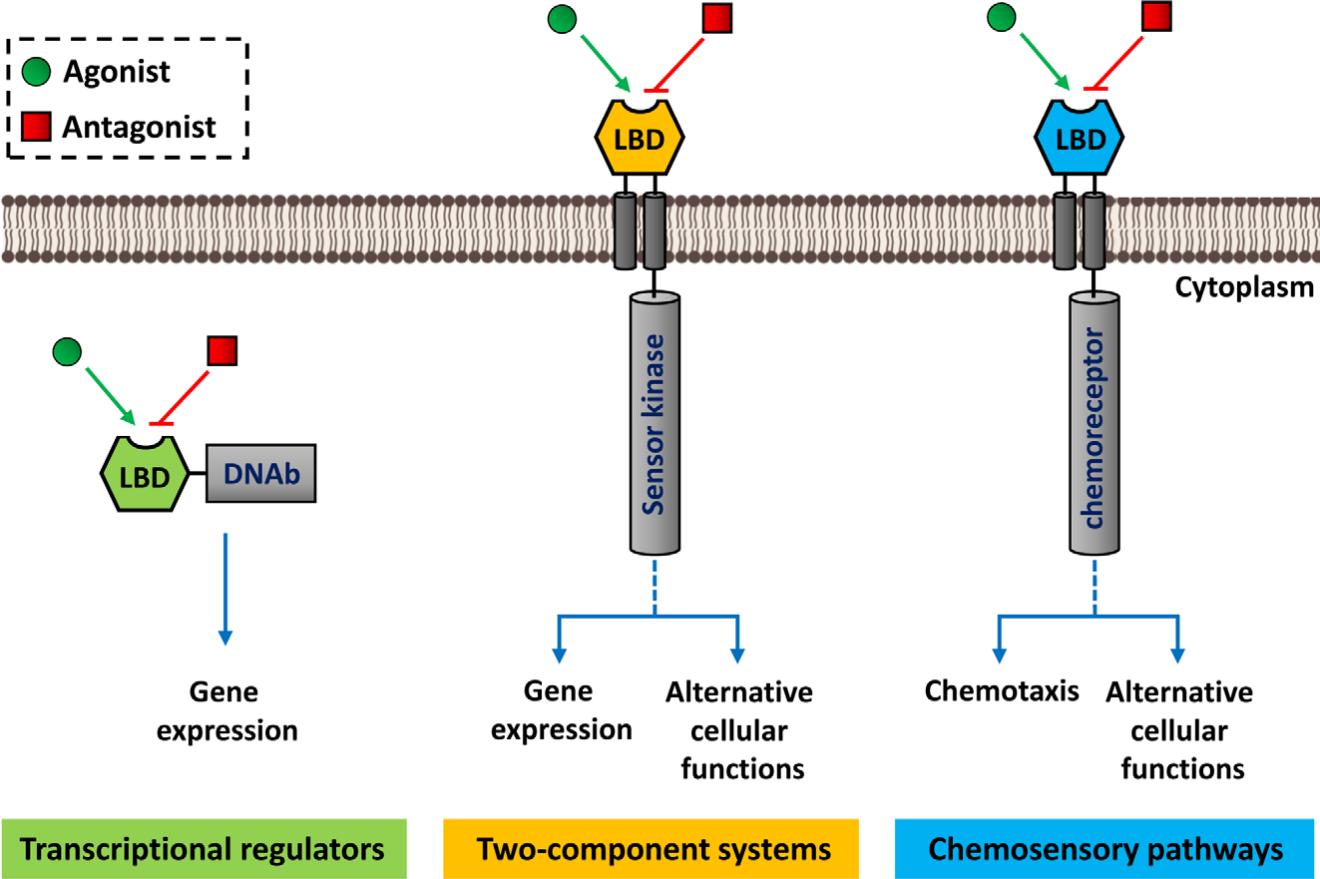
Agonist‐ and antagonist‐mediated modulation of the activity of transcriptional regulators, two component systems and chemosensory pathways. Agonist and antagonists bind to the ligand binding domain (LBD) of sensor proteins inducing or repressing downstream signalling, processes that are represented by triangular and flat arrowheads, respectively. DNAb, DNA‐binding domain.

The canonical topology of sensor proteins like sensor histidine kinases or chemoreceptors consists of an extracytoplasmic ligand‐binding domain (LBD) and a cytosolic domain that is involved in processing the molecular stimulus created by signal binding (Fig. 2). Whereas the cytosolic domains of sensor kinases and chemoreceptors are generally rather conserved in their sequence, there is an enormous diversity among the LBDs. This diversity is reflected in (i) the presence of many different LBD types, such as 80 in chemoreceptors (Ortega *et al*., 2017) and (ii) by an important sequence divergence with individual LBD families (Gavira *et al*., 2020). These notions, thus, imply that a specific interference with signal transduction systems can be best achieved by targeting the specific part of sensor proteins, namely the LBD (Fig. 2).

There is now a significant body of evidence for the existence of naturally occurring signal antagonists (Busch *et al*., 2007; Martin‐Mora *et al*., 2018; Johnson *et al*., 2021), which are compounds that bind to the same site as the signal molecules but fail to induce downstream signalling (Fig. 2) – a phenomenon likely due to different conformational changes induced by signal and signal antagonist binding (Koh *et al*., 2016). Antagonists and signal molecules are frequently structurally similar, but the molecular features that determine their agonistic or antagonistic function are currently unclear. However, advances in computational biology have permitted that protein ligands can be identified by high‐throughput *in silico* molecular docking approaches of compound libraries to three‐dimensional structures of target proteins.

Virulence properties are frequently controlled by quorum‐sensing mechanisms, and the receptors for quorum‐sensing signals are major targets for anti‐infective therapy. A number of *in silico* docking studies have reported the identification of quorum‐sensing antagonists, as exemplified by (Gnanendra *et al*., 2013; Nandi, 2016; Vetrivel *et al*., 2021). This approach is based on the availability of high‐resolution 3D target protein structures, and their absence is a major limitation of this technique. However, the possibility of generating highly precise 3D protein models using the AlphaFold technology has started to revolutionize biology (Jumper *et al*., 2021; Tunyasuvunakool *et al*., 2021). These precise models will facilitate the resolution of the crystallographic phase problem using molecular replacement techniques leading to a more rapid resolution of experimental structures or, alternatively, AlphaFold‐created models will be used directly for *in silico* ligand screening. Such approaches will facilitate the identification of LBD ligands, of which their either agonistic or antagonistic effect on signal transduction needs to be determined experimentally.

A number of studies have reported the identification of inhibitors that target the conserved (cytosolic) part of sensor proteins such as the autokinase domain of sensor kinases (Hirakawa *et al*., 2020). A major disadvantage of these inhibitors is the necessity to cross the bacterial membrane to reach their targets. As a consequence, most of these compounds are rather hydrophobic that diffuse across the membrane, and in several occasions this hydrophobicity was associated with a reduced specificity of the inhibitor (Hirakawa *et al*., 2020). In addition, hydrophobic compounds may have the potential to integrate into human membranes that may cause undesired side‐effects. In this context, the specific targeting of extracytoplasmic sensor domains appears to be advantageous.

## Concluding remarks and perspectives

Currently, our main weapons to fight pathogenic bacteria are antibiotics that either kill or slow down bacterial growth. However, the rise of AMR infections is alarming, and the identification of new chemicals to combat pathogenic microorganisms is urgently needed. This aspect is reflected in the fact that (i) no novel classes of antibiotics effective against Gram‐negative bacteria have been discovered in more than half a century (Hutchings *et al*., 2019) and (ii) only ~ 40 antibacterial molecules are currently in clinical trials; a number largely inferior to the approximately 4000 immuno‐oncology agents that are currently under development (Xin Yu *et al*., 2019). However, advances in multidisciplinary approaches including genomics, metagenomics, proteomics, synthetic biology, high‐throughput culturing methods, bioinformatics and analytical chemistry are contributing to reinvigorate antibiotics research, which will benefit from exploring unconventional ecological niches and under‐investigated microbial taxonomic groups. In addition, anti‐infective therapy is (re‐) emerging as an alternative approach to antibiotics. There is significant promise in targeting sensor domains of signal transduction proteins that are required for full virulence during host infection by a knowledge‐based application of key signal molecules or by signal antagonists. The identification of these molecules will be facilitated by an increasing computing power and precision of molecular docking approaches. Notably, a major bottleneck of such approaches, namely the absence of high‐resolution target structures, will be less important due to the possibilities offered by the AlphaFold technology.

The combined action of antibiotics and antivirulence drugs has been suggested to enable a greater control of pathogenic microorganisms with a reduced occurrence of AMR (Miethke *et al*., 2021). These strategies are in line with the action plans introduced by the World Health Organization (WHO, 2015) and the European Commission (EC, 2017) to combat the rising of AMR infections at a global scale.

## Funding information

This work was supported through grants from the CSIC to MAM (PIE‐202040I003), the Spanish Ministry for Science and Innovation to MAM (PID2019‐103972GA‐I00), the Junta de Andalucía (P18‐FR‐1621) and Spanish Ministry of Economy and Competitiveness (BIO2016‐76779‐P) to TK.

## Conflict of interest

The authors declare that there is no conflict of interest.
